# Evaluation of *LOXL1* polymorphisms in exfoliation syndrome in the Uygur population

**Published:** 2011-06-28

**Authors:** Mayinu, Xueyi Chen

**Affiliations:** Department of Ophthalmology, the First Affiliated Hospital of Xinjiang Medical University, Urumchi, Xinjiang Uygur Autonomous Region, China

## Abstract

**Purpose:**

In Uygur populations, exfoliation syndrome (XFS) and exfoliation glaucoma (XFG) occurred at a high frequency. In this study, we evaluate the association profiles of the lysyl oxidase-like 1 (*LOXL1*) gene polymorphisms with XFS in the Uygur population.

**Methods:**

Sixty-four unrelated Uygur patients with XFS and 127 Uygur control subjects were included in this study. Genotypes of the three single nucleotide polymorphisms (SNPs) of *LOXL1* (rs1048661, rs2165241, and rs3825942) were analyzed by direct sequencing, and a case-control association study was performed.

**Results:**

The three SNPs were significantly associated with XFS and XFG individually. The G allele of rs1048661 (OR [95%CI]: 1.92 [1.14–3.22]), G of rs3825942 (OR [95%CI]: 4.86 [2.02–11.68]), and T of rs2165241 (OR [95%CI]: 3.98 [2.54–6.25]) were risk alleles for the disorder. The genotypes GG for rs1048661 (OR [95%CI]: 2.13 [1.14–3.97]), GG for rs3825942 (OR [95%CI]: 5.68 [2.28–14.17]), and TT for rs2165241 (OR [95%CI]: 6.13 [2.68–14.01]) were risk genotypes for the disease. The haplotypes G-G for the SNPs rs1048661and rs3825942, G-T for the SNPs rs1048661 and rs2165241, and SNPs rs3825942 and rs2165241 were found to be significantly associated with XFS/G. The haplotypes G-G-T for the three SNPs were determined to be significantly associated with XFS/G. There were significant differences of the allelic and genotypic proportion in different gender/age patients and controls for all three SNPs. T allele of rs2165241 and G of rs3825942 were risk alleles for the disorder in both the male and female groups. G allele of rs1048661 was a risk allele for the disorder in the below 65-year-old group. T of rs2165241 was a risk allele for the disorder in both age groups. G of rs3825942 was a risk allele for the disorder in the over 65-year-old group. The genotypes also showed significant differences in the below 65-year-old group of rs1048661, both groups of rs2165241, and over 65-year-old group of rs3825942.

**Conclusions:**

*LOXL1* is a susceptibility gene of XFS/XFG in Uygur populations. The risk alleles of rs1048661, rs3825942 and rs2165241 in Uygur subjects were found to be similar to those of populations in Iceland and the United States and different from Han populations in China. The genotypic and allelic distributions of these SNPs are similar between XFS and XFG.

## Introduction

Exfoliation syndrome (XFS) is an age-related, systemic, elastic microfibrillopathy affecting both intraocularand extraocular tissues [1]. The clinical features: white flake-like material on the anterior lens surface, the pupillary border, trabecular meshwork, zonula, ciliary body, and other anterior segment structures etc were initially described by Lindberg in 1917 [2]. Exfoliation glaucoma (XFG) is characterized by rapid progression, high resistance to medical therapy, and poor prognosis. XFS can not only lead to severe chronic open-angle glaucoma but also to acceleration of cataract formation, lens subluxation, angle-closure glaucoma, and severe complications at the time of cataract extraction, such as zonular dialysis, capsular rupture, and vitreous loss [2-5]. Especially in the Uygur populations in Xinjiang, because of lack of medicines and awareness of the disease, XFS/G patients go to the hospital when they have almost lost their visual acuity.

Epidemiological studies have shown that the prevalence of XFS varies greatly among ethnic groups, with a prevalence of 28% in Iceland, 20% in Finland, a little lower in Norway and Switzerland [6], and 1.6~6.3% in north America and western Europe. The highest incidence is in the Scandinavian peninsula [7], while prevalence is 0% in the Greenland Inuit population [8,9]. It can affect 10%–20% of the elderly populations, so the elderly populations and glaucoma patients have a higher incidence [10]. In Asian populations, the prevalence is 3.01%–6.28% in Indians aged over 40 years [11,12], 3.4% in Japanese aged over 50 years [13], 0.4% in Hong Kong Chinese aged over 60 years [14], 0.2% and 0.7% in Singaporean Chinese aged over 40 and over 60 years [15], 3.3% in the Han people in China [16], 5.1% in the Kashi Uygur [17], and 2.2% and 9.5% in the Kuche Uygur aged over 60 and 80 years, respectively [18].

According to studies worldwide, there is evidence showing that genetic factors may play an important role in the pathogenesis of XFS [19]. Recently, Thorleifsson et al. [20] performed a genome-wide association study and identified a strong association of XFG with three single nucleotide polymorphisms (SNPs) in the lysyl oxidase-like 1 gene (*LOXL1*) on 15q24.1. They identified one intronic SNP (rs2165241) and two nonsynonymous coding SNPs (rs3825942 and rs1048661) with significant disease association in Icelandic and Swedish individuals. This association was recently replicated in the midwestern United States [21], Australian populations [22], Indian populations [23], Japanese populations [24], and Chinese populations [25]. XFS has a high prevalence in Uygur populations [17,18]. Therefore it is logically important to perform a case-control study using another ethnic population like the Uygur minority in China who are different from the Han people, to uncover the pathogenesis of XFS/G in Uygur patients.

Xinjiang lies in northwest China, bordering Gansu and Qinghai provinces to the southeast and the Tibet Autonomous Region to the south, and shares a 5,000-km border with eight countries. It is the largest Chinese administrative division, with a total population of nearly 21.56 million, and it is home to 47 of China's 56 ethnic groups, such as Uygur (47.47%), Han (37.58%), and Kazak (7.3%). We recruited our Uygur case and control subjects from Kuche and Kashi, located in the south of the Tianshan Mountains, where the urban population is mainly Uygur.

## Methods

### Study subjects

The diagnostic criterion for XFS was the existence of exfoliation material on the anterior lens capsule or on the pupil margin in either eye with dilation of the pupils. Patients with intraocular pressure (IOP) of less than 21 mmHg and no clinical evidence of glaucomatous optic neuropathy were classified as XFS. XFG was diagnosed if the patient had the above characteristics of exfoliation syndrome and all of the following features: (1) IOP ≥22 mmHg in either eye; (2) glaucomatous changes on the optic disc, defined as cup to disc ratio >0.7 in either eye or an asymmetric cup to disc ratio of >0.2 or notching of the disc rim; and (3) characteristic glaucomatous visual field loss [26]. Patients with other causes of secondary glaucoma, such as uveitis, pigment dispersion syndrome, and iridocorneal endothelial syndrome, were excluded. The patients were recruited from Kashi and Kuche Uygur people over 45 years of age. The controls were enrolled by the following criteria: (1) no signs of XFS or XFG, (2) no glaucomatous changes on the optic disc, (3) normal visual field and intraocular pressure, (4) no family history of glaucoma, and (5) no other eye diseases except mild refractive errors. All study subjects were unrelated. All study subjects received comprehensive ophthalmic examinations.

The research protocol was approved by the Ethics Committee for Human Research of the First Affiliated Hospital of Xinjiang Medical University, China. Informed consent was obtained from all participants after explaining the objective and nature of the study. The study was conducted in accordance with the Declaration of Helsinki.

Peripheral blood samples (2–3 ml) were collected from each subject by venipuncture and genomic DNA was extracted from whole blood by using a Genomic DNA Extraction Kit, (The Beijing Genomics Institute, Beijing, China). The three SNPs (rs1048661, rs3825942, and rs2165241) in the *LOXL1* gene, in accordance with previous reports, were amplified by PCR and directly sequenced [20,23]. Two sets of primers were used for amplification by PCR (Table 1). The PCR protocol was as follows: For rs2165241: initial denaturation at 94 °C/5 min, followed by 10 cycles (94 °C/30 s, 65–60 °C touchdown for 1 min with 0.5 °C decrement, 72 °C/45 s), 30 cycles (94 °C/30 s, 60 °C/1 min, 72 °C/45 s), and a final extension at 72 °C/1 min. For rs1048661 and rs3825942: initial denaturation at 94 °C for 5 min followed by denaturation at 94 °C for 30 s, annealing at 58–55 °C touchdown for 30 s with 0.5 °C decrement for the first six cycles, extension at 72 °C for 45 s for 35 cycles, and a final extension of 72 °C for 5 min.

**Table 1:** Primer sequences for PCR for SNPs of *LOXL1*.

**Amplicon**	**Primer sequences**	**Size (bp)**
rs1048661, rs3825942*	F: 5′-ATTCGGCTTTGGCCAGGT-3′	178
	R: 5′-GAACTGCTGCGGGTAGGA-3′	
rs2165241	F: 5′-CTCTAGGGCCCCTTGGAGAAT-3′	321
	R: 5′-GGCCAGAGGTCTGCTAAGCAC-3′	

Two sets of primers were used for PCR for the three SNPs. The asterisk indicates that the same set of primers was used for rs1048661 and rs3825942.

**Figure 1:**
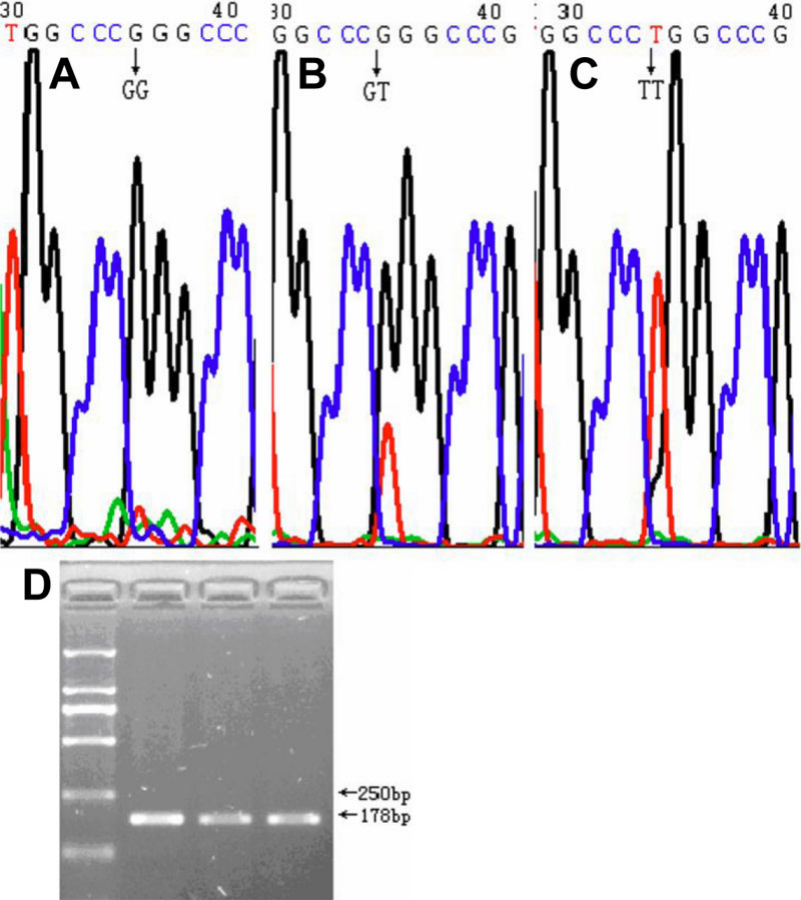
Representative chromatograms displaying and the PCR electrophoresis image for the SNP rs1048661. **A**, **B**, **C**: The three different genotypes of the SNP rs1048661. **D**: The PCR electrophoresis image for the SNP rs1048661.

**Figure 2:**
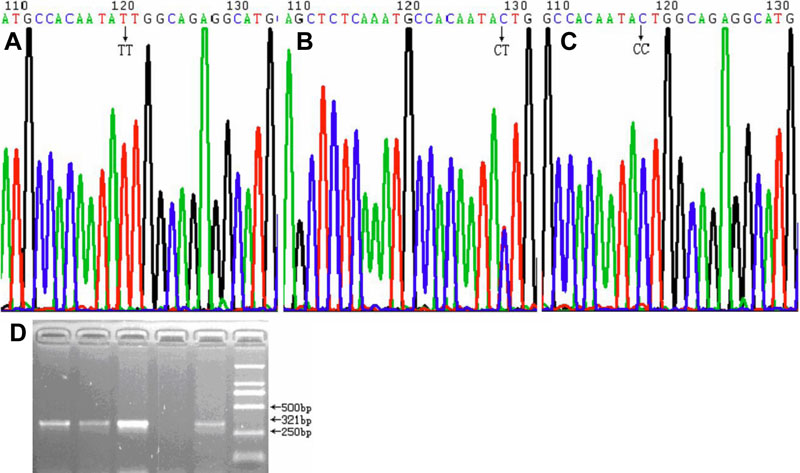
Representative chromatograms displaying and the PCR electrophoresis image for the SNP rs2165241. **A**, **B**, **C**: The three different genotypes of the SNP rs2165241. **D**: The PCR electrophoresis image for the SNP rs2165241.

**Figure 3:**
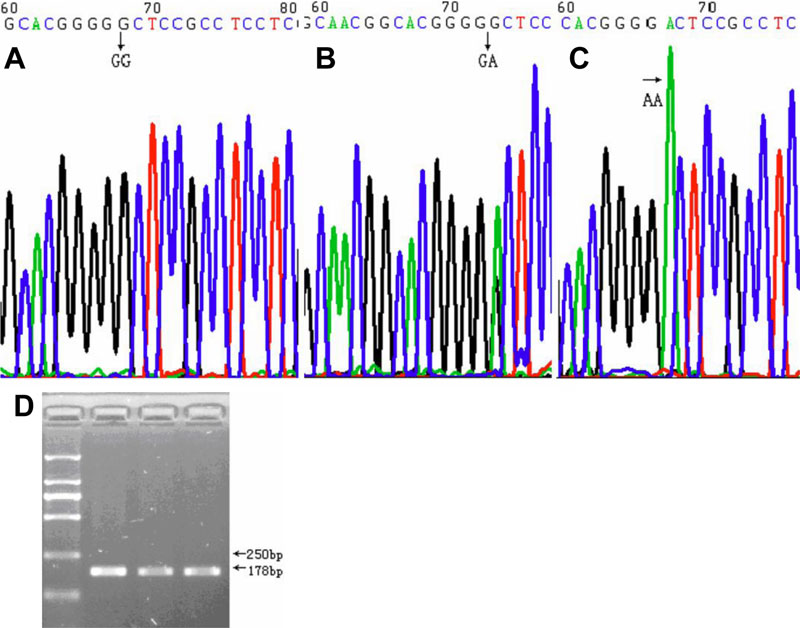
Representative chromatograms displaying and the PCR electrophoresis image for the SNP rs3825942. **A**, **B**, **C**: The three different genotypes of the SNP rs3825942. **D**: The PCR electrophoresis image for the SNP rs3825942

Genotypes of the three *LOXL1* SNPs were determined by direct DNA sequencing, using BigDye Terminator v3.1 Kit (Applied Biosystems, Foster City, CA) in a 3730XL capillary sequencer (Applied Biosystems). The sequences were analyzed by sequencing analysis software Chromas (Technelysium Pty Ltd., Queensland, Australia). Representative chromatograms displaying these three SNPs are provided in Figure 1 (rs1048661), Figure 2 (rs2165241), and Figure 3 (rs3825942).

### Statistical analysis

Statistical analysis was performed using SPSS v13.0 software package (SPSS Inc., Chicago, IL). Hardy–Weinberg equilibrium (HWE) analysis was tested by using the χ^2^ test in SAS/Genetics v9.1 (SAS Institute Inc., Cary, NC). The comparison of allelic and genotypic frequencies between the patient and control groups as well as haplotype association analysis were performed using a standard χ^2^ test with a p-value <0.05 considered statistically significant. Relative risk association was estimated by calculating odds ratios (OR) along with 95% confidence intervals (CI).

## Results

A total of 64 Uygur patients with exfoliation syndrome, including 7 cases of XFG, 4 cases of XFS with high IOP, and 53 cases of XFS without glaucoma, as well as 127 Uygur control individuals, were recruited into this study. The age at recruitment was 52–95 years (mean age 70.5±9.3 years) in the patient group, and45–84 years (mean age 63.7±10.0 years) in the control group. The gender distribution between the patient and control groups was not significantly different (χ^2^=0.01, p=0.97), with 41 (64.1%) males and 23 (35.9%) females in the patient group and 81 (63.8%) males and 46 (36.2%) females in the control group. The clinical features of the patients are shown in Table 2. Eleven of the 64 XFG patients had severe glaucoma and high IOP. Only one subject had trabeculectomy and iridotomy, and two were treated with medicine.

**Table 2:** Clinical features of the patients with XFS/XFG.

** **	**Total (n=64)**		
**Clinical features**	**XFG (n=7)**	**XFS with high IOP (n=4)**	**XFS (n=53)**
Age at recruitment (mean±SD)	67.00±7.16	67.75±10.01	71.15±9.53
Range	58–76	60–81	52–95
Gender (male/female)	5/2	3/1	33/20
History of trabeculectomy	n=1	n=0	n=0
History of laser trabeculoplasty	n=0	n=0	n=0
History of laser iridotomy	n=1	n=0	n=0
Treated with medicine	n=2	n=0	n=0

A total of 64 patients with exfoliation syndrome were recruited into this study, including 7 cases with XFG and 53 cases with XFS without glaucoma. Only one of the 7 XFG patients had trabeculectomy and laser iridotomy and 2 patients were treated with medicine.

The genotype distribution of SNP rs1048661 (p=0.84 for patients and p=0.68 for controls), rs3825942 (p=0.69 for patients and p=0.12 for controls), and rs2165241 (p=0.37 for patients and p=0.24 for controls) followed the HWE in patients and controls. Allelic association analysis showed that there were significant differences in the allelic distributions between the two groups for the three SNPs (Table 3). The frequency of allele G of rs1048661 was higher in the patient group than in the control group (p=0.013, OR=1.92, 95% CI: 1.14–3.22). The frequency of allele T of rs2165241 was significantly higher in the patient group than in the control group (p=0.000, OR=3.98, 95% CI: 2.54–6.25). The frequency of allele G of rs3825942 was significantly higher in the patient group than in the control group (p=0.000, OR=4.86, 95% CI: 2.02–11.68). The genotypic frequencies for each of the three SNPs were also compared between patients and controls (Table 3). The frequency of genotype GG at SNP rs1048661 was higher in patients than in controls (p=0.016, OR=2.13, 95% CI: 1.14–3.97), and the frequencies of TT genotypes were not significantly different between the two groups. The frequency of genotype TT at SNP rs2165241 was significantly higher in the patient group than in the control group (p=0.000, OR=6.13, 95% CI: 2.68–14.01), and the frequency of CC genotypes was significantly lower in the patients than in the controls. The frequency of genotype GG at SNP rs3825942 was significantly higher in the patient group than in the control group (p=0.000, OR=5.68, 95% CI: 2.28–14.17), and the frequencies of AA genotypes were not significantly different between the two groups.

**Table 3:** Allele and genotype association analysis for the three SNPs of *LOXL1*.

**SNP**	**XFS/XFG**	**Control**	**χ^2^**	**p value**	**OR (95%CI)**
rs1048661					
**Allele**					
G	104 (0.81)	176 (0.69)	6.22	0.013	1.92 (1.14–3.22)
T	24 (0.19)	78 (0.31)			
**Genotype**					
GG	42 (0.66)	60 (0.47)	5.78	0.016	2.13 (1.14–3.97)
GT	20 (0.31)	56 (0.44)			
TT	2 (0.03)	11 (0.09)	2.06	0.152	0.34 (0.07–1.58)
Total	64	127	6.37	0.041	NA
rs2165241					
**Allele**					
T	72 (0.56)	62 (0.24)	37.89	0.000	3.98 (2.54–6.25)
C	56 (0.44)	192 (0.76)			
**Genotype**					
TT	22 (0.34)	10 (0.08)	21.43	0.000	6.13 (2.68–14.01)
CT	28 (0.44)	42 (0.33)			
CC	14 (0.22)	75 (0.59)	23.64	0.000	0.19 (0.10–0.39)
Total	64	127	31.79	0.000	NA
rs3825942					
**Allele**					
G	122 (0.95)	205 (0.81)	14.73	0.000	4.86 (2.02–11.68)
A	6 (0.05)	49 (0.19)			
**Genotype**					
GG	58 (0.91)	80 (0.63)	16.21	0.000	5.68 (2.28–14.17)
GA	6 (0.09)	45 (0.35)			
AA	0 (0.00)	2 (0.02)	1.02	0.313	NA
Total	64	127	16.33	0.000	NA

There were significant differences for the allelic proportion between the patient and control groups for all three SNPs. G allele of rs1048661, G of rs3825942, and T of rs2165241 were risk alleles for the disorder. The genotypes GG for rs1048661, GG for rs3825942, and TT for rs2165241 were risk genotypes for the disease. Total indicates the general test of association in the 2-by-3 table of disease-by-genotype. NA: not available. The asterisks indicates the OR values and p values derived from comparison of the specific genotype with all the others, i.e., GG versus GT+TT at rs1048661, TT versus CT+CT at rs2165241.

Haplotypes defined by the three SNPs were analyzed (Table 4). For the SNPs rs1048661 and rs3825942, three haplotypes were observed. The haplotype G-G (p=0.006, OR=3.81, 95% CI: 1.40–10.34) was identified to be significantly associated with the patient group. For SNPs rs1048661 and rs2165241, four haplotypes were observed, among which the haplotype G-T was significantly associated with the patient group (p=0.000, OR=3.66, 95% CI: 1.99–6.90). For the SNPs rs3825942 and rs2165241, three haplotypes were observed, with the haplotype G-T showing a significantly higher frequency in the patients than in the controls (p=0.000, OR=3.38, 95% CI: 1.80–6.34). The haplotypes for the three SNPs were also estimated. The haplotype G-G-T had a significantly higher frequency in the patients than in the controls (p=0.000, OR=3.58, 95% CI: 1.90–6.76).

**Table 4:** Haplotype Association analysis between the *LOXL1* SNPs and XFS/XFG.

**Haplotype**			**Proportion**		**χ^2^**	**p value**	**OR (95%CI)**
			**Case**	**Control**			
rs1048661	rs3825942						
T	G		0.047	0.181	6.52	0.011	0.22 (0.06–0.77)
G	G		0.922	0.756	7.66	0.006	3.81(1.40–10.34)
G	A		0.031	0.063	0.86	0.353	0.48 (0.10–2.33)
Total					7.90	0.019	NA
rs1048661	rs2165241						
T	C		0.031	0.157	6.65	0.010	0.17 (0.04–0.76)
G	T		0.563	0.260	16.89	0.000	3.66 (1.99–6.90)
T	T		0.016	0.024	0.13	0.716	0.66 (0.07–6.44)
G	C		0.391	0.559	4.83	0.028	0.51 (0.27–0.93)
Total					19.21	0.000	NA
rs3825942	rs2165241						
G	C		0.406	0.660	11.35	0.001	0.35 (0.19–0.65)
A	T		0.016	0.008	0.25	0.619	2.00(0.12–32.51)
G	T		0.563	0.280	15.00	0.000	3.38 (1.80–6.34)
A	C		0.016	0.055	1.65	0.198	0.27 (0.03–2.26)
Total					16.06	0.001	NA
rs1048661	rs3825942	rs2165241					
T	G	C	0.031	0.157	6.65	0.010	0.17 (0.04–0.76)
G	G	C	0.375	0.504	2.85	0.092	0.59 (0.32–1.09)
G	G	T	0.547	0.252	16.25	0.000	3.58 (1.90–6.76)
T	G	T	0.016	0.024	0.13	0.716	0.66 (0.07–6.44)
G	A	C	0.016	0.055	1.65	0.198	0.27 (0.03–2.26)
G	A	T	0.016	0.008	0.25	0.619	2.00(0.12–32.51)
Total					19.93	0.001	NA

The haplotypes G-G for the SNPs rs1048661 and rs3825942, G-T for the SNPs rs1048661 and rs2165241, and G-T for the SNPs rs3825942 and rs2165241 were identified to be significantly associated with XFS/G. The haplotype G-G-T for the three SNPs was identified to be significantly associated with XFS/G. Total indicates omnibus tests, while the others indicate the haplotype-specific tests. The OR values were obtained from comparison of each individual haplotype with all the other haplotypes, i.e., T-G versus G-G + G-A for the SNPs rs1048661 and rs3825942, etc. NA: not available.

Allelic and genotypic association analysis by gender showed that there are significant differences in the allelic and genotypic distributions between the two groups for the three SNPs (Table 5). The G allele of rs1048661 was not significantly different in either the male group or the female group. At rs2165241 frequency of the T allele was higher in male patients than in male controls (p=0.001, OR=2.51, 95% CI: 1.44–4.39) and was significantly higher in female patients than in female controls as well. (p=0.000, OR=9.75, 95% CI: 4.31–22.07). At rs3825942 frequency of the G allele was higher in male patients than in male controls (p=0.005, OR=3.79, 95% CI: 1.42–10.14) and was significantly higher in female patients than in female controls (p=0.007, OR=10.2, 95% CI: 1.31–79.26). The genotypic frequencies for each of the three SNPs were also compared between the patient and control groups by gender (Table 5). The frequency of genotype GG at SNP rs1048661 was not significantly different in either the male group or the female group. The frequencies of genotype TT at SNP rs2165241 and GG at SNP rs3825942 were significantly higher in the patients than in the controls in both gender groups.

**Table 5:** Allele and genotype association analysis between the *LOXL1* SNPs and different gender of XFS/XFG.

** **	** **	** **	**Genotype count (proportion)**					**Allele count (proportion)**				
**Gender**	**Group**	rs1048661** (n)**	**TT**	**GT**	**GG**	**χ^2^**	**p value**	**G**	**T**	**χ^2^**	**p value**	**OR (95%CI)**
Male	XFS/XFG	41	1 (0.02)	14 (0.34)	26 (0.63)	4.08	0.130	66 (0.80)	16 (0.20)	3.92	0.048	1.90 (1.00–3.59)
	Control	81	10 (0.12)	31 (0.38)	40 (0.49)			111 (0.69)	51 (0.31)			
Female	XFS/XFG	23	1 (0.04)	6 (0.26)	16 (0.70)	4.98	0.083	38 (0.83)	8 (0.17)	2.32	0.128	1.97 (0.82–4.78)
	Control	46	1 (0.02)	25 (0.54)	20 (0.43)			65 (0.71)	27 (0.29)			
** **	** **	** **	**Genotype count (proportion)**					**Allele count (proportion)**				
**Gender**	**Group**	rs2165241** (n)**	**CC**	**CT**	**TT**	**χ^2^**	**p value**	**T**	**C**	**χ^2^**	**p value**	**OR (95%CI)**
Male	XFS/XFG	41	12 (0.29)	19 (0.46)	10 (0.24)	9.45	0.009	39 (0.48)	43 (0.52)	10.8	0.001	2.51 (1.44–4.39)
	Control	81	46 (0.57)	27 (0.33)	8 (0.10)			43 (0.27)	119 (0.73)			
Female	XFS/XFG	23	2 (0.09)	9 (0.39)	12 (0.52)	27.6	0.000	33 (0.72)	13 (0.28)	34.1	0.000	9.75 (4.31–22.07)
	Control	46	29 (0.63)	15 (0.33)	2 (0.04)			19 (0.21)	73 (0.79)			
** **	** **	** **	**Genotype count (proportion)**					**Allele count (proportion)**				
**Gender**	**Group**	rs3825942** (n)**	**GG**	**GA**	**AA**	**χ^2^**	**p value**	**G**	**A**	**χ^2^**	**p value**	**OR (95%CI)**
Male	XFS/XFG	41	36 (0.88)	5 (0.12)	0 (0.00)	8.41	0.015	77 (0.94)	5 (0.06)	7.89	0.005	3.79 (1.42–10.14)
	Control	81	51 (0.63)	28 (0.35)	2 (0.02)			130 (0.80)	32 (0.20)			
Female	XFS/XFG	23	22 (0.96)	1 (0.04)	0 (0.00)	8.46	0.004	45 (0.98)	1 (0.02)	7.19	0.007	10.2 (1.31–79.26)
	Control	46	29 (0.63)	17 (0.37)	0 (0.00)			75 (0.82)	17 (0.18)			

There were significant differences in allelic and genotypic proportion between the genders in the patient and control groups for the SNPs rs2165241 and rs3825942. T allele of rs2165241 and G of rs3825942 were risk alleles for the disorder for both the male and female groups. Total indicates the general test of association in the 2-by-3 table of disease-by-genotype.

Allelic and genotypic association analysis in different aged groups showed that there were significant differences in the allelic and genotypic distributions between the two groups for the three SNPs (Table 6). At rs1048661 the frequency of the G allele was higher in the patients than in the controls in the below 65-year-old group (p=0.011, OR=3.86, 95% CI: 1.29–11.58). At rs2165241 the frequency of the T allele was significantly higher in the patients than in the controls in both aged groups. (≤65:p=0.000, OR=4.41, 95% CI: 2.09–9.30; >65: p=0.000, OR=3.93, 95% CI: 2.18–7.10). At rs3825942 the frequency of the G allele was significantly higher in the patients than in the controls in the over 65-year-old group. (p=0.000, OR=9.04, 95% CI: 2.66–30.71). The genotypic frequencies for each of the three SNPs were also compared between patients and controls in the different age groups (Table 6). The frequency of genotype GG at SNP rs1048661 was significantly higher in the patients than in the controls in the below 65-year-old age group. The frequency of genotype TT at SNP rs2165241 was significantly higher the patients than in the controls in both aged groups. GG at SNP rs3825942 was significantly higher in patients than in controls in the over 65-year-old group.

**Table 6:** Allele and genotype association analysis between the *LOXL1* SNPs and different aged XFS/XFG.

** **	** **	** **	**Genotype count (proportion)**					**Allele count (proportion)**				
** Years**	**Group**	rs1048661** (n)**	**TT**	**GT**	**GG**	**χ^2^**	**p value**	**G**	**T**	**χ^2^**	**p value**	**OR (95%CI)**
≤65	XFS/XFG	20	0 (0.00)	4 (0.20)	16 (0.80)	7.32	0.026	36 (0.90)	4 (0.10)	6.48	0.011	3.86 (1.29–11.58)
	Control	65	4 (0.06)	31(0.48)	30 (0.46)			91 (0.70)	39 (0.30)			
>65	XFS/XFG	44	2 (0.05)	16(0.36)	26 (0.59)	2.04	0.360	68 (0.77)	20 (0.23)	1.95	0.163	1.56 (0.83–2.92)
	Control	62	7 (0.11)	25 (0.40)	30 (0.48)			85 (0.69)	39 (0.31)			
** **	** **	** **	**Genotype count (proportion)**					**Allele count (proportion)**				
**Years**	**Group**	rs2165241** (n)**	**CC**	**CT**	**TT**	**χ^2^**	**p value**	**T**	**C**	**χ^2^**	**p value**	**OR (95%CI)**
≤65	XFS/XFG	20	2 (0.10)	12 (0.60)	6 (0.30)	16.3	0.000	24 (0.60)	16 (0.40)	16.5	0.000	4.41 (2.09–9.30)
	Control	65	40 (0.62)	17 (0.26)	8 (0.12)			33 (0.25)	97 (0.75)			
>65	XFS/XFG	44	12 (0.27)	16 (0.36)	16 (0.36)	21.7	0.000	48 (0.55)	40 (0.45)	21.6	0.000	3.93 (2.18–7.10)
	Control	62	35 (0.56)	25 (0.40)	2 (0.03)			29 (0.23)	95 (0.77)			
** **	** **	** **	**Genotype count (proportion)**					**Allele count (proportion)**				
**Years**	**Group**	rs3825942** (n)**	**GG**	**GA**	**AA**	**χ^2^**	**p value**	**G**	**A**	**χ^2^**	**p value**	**OR (95%CI)**
≤65	XFS/XFG	20	17 (0.85)	3 (0.15)	0 (0.00)	1.61	0.204	37 (0.93)	3 (0.07)	1.38	0.241	2.11 (0.59–7.54)
	Control	65	46 (0.71)	19 (0.29)	0 (0.00)			111 (0.85)	19 (0.15)			
>65	XFS/XFG	44	41 (0.93)	3 (0.07)	0 (0.00)	18.4	0.000	85 (0.97)	3 (0.03)	16.9	0.000	9.04 (2.66–30.71)
	Control	62	34 (0.55)	26 (0.42)	2 (0.03)			94 (0.76)	30 (0.24)			

There were significant differences of the allelic and genotypic proportion in different aged patient and control groups for all three SNPs. G allele of rs1048661 was a risk allele for the disorder in the below 65-year-old group. T of rs2165241 was a risk allele for the disorder in both aged groups. G of rs3825942 was a risk allele for the disorder in the over 65-year-old group. The genotypes also showed significant differences in the below 65-year-old group of rs1048661, both aged groups of rs2165241, and over 65-year-old group of rs3825942. Total indicates the general test of association in the 2-by-3 table of disease-by-genotype.

## Discussion

This is the first study to show that the polymorphisms of *LOXL1* are associated with Uygur patients with XFS/G. These results are important in understanding the role of *LOXL1* polymorphisms in the pathogenesis of XFS/G in Uygur patients. The association between *LOXL1* and XFS/XFG in the Han population has rarely been reported except in one recent report from Beijing Tongren Eye Center [25]. In this study, we recruited samples of XFS/XFG patients from Kuche and Kashi which are inhabited mostly by Uygur people. Three major *LOXL1* SNPs were found to be significantly associated with XFS/XFG, even though the sample size was small at 64. Hence, the effect sizes of these SNPs in the Uygur population were large, and 64 samples provided a good statistical power to detect significant association. In Xinjiang, it is usually difficult to recruit a large sample of XFS/XFG throughout the autonomous region to ensure frequency matching because of the long distance between cities.

The reason for the higher prevalence in Uygur individuals than in Han individuals is still unknown [16-18]. In this study, we found that all at-risk alleles and genotypes of the *LOXL1* SNPs in the Uygur population are different from the Han population but similar to Iceland [20] and the United States [27]. Compared to the frequency of the G allele at rs1048661, the frequency of the T allele at rs2165241 was significantly higher in the patients than in the controls (p=0.000, OR=3.98, 95% CI: 2.54–6.25), and the G allele at rs3825942 was also significantly higher in the patients than in the controls (p=0.000, OR=4.86, 95% CI: 2.02–11.68).

Such a discrepancy might not be fully explained by the ethnic difference in the frequencies of the at-risk alleles of the *LOXL1* SNPs (Table 7), and other genetic and/or environmental factors might be involved in the development of the disorder.

**Table 7:** Risk alleles and MAF for the three SNPs of *LOXL1* in different populations.

** **	rs1048661** (G/T)**		rs3825942** (G/A)**		rs2165241** (T/C)**		** **
**Population**	**Risk allele**	**MAF**	**Risk allele**	**MAF**	**Risk allele**	**MAF**	**Reference **
Iceland	G	0.349(T)	G	0.153 (A)	T	0.473 (T)	[20]
Sweden	G	0.318 (T)	G	0.121 (A)	T	0.465 (C)	
Austria	G	0.329 (T)	G	0.183 (A)	NA	NA	[28]
United States	G	0.335 (T)	G	0.156 (A)	T	0.487 (T)	[27]
United States	G	0.297 (T)	G	0.202 (A)	T	0.448 (T)	[29]
United States	G	0.400 (T)	G	0.120 (A)	NA	NA	[30]
United States	G	0.281 (T)	G	0.205 (A)	T	0.456 (T)	[31]
Germany and Italy	G	0.348 (T)	G	0.149 (A)	T	0.488 (T)	[32]
Australia	G	NA	G	NA	NA	NA	[22]
refSNP (European)	NA	0.040 (T)	NA	0.172 (T)	NA	0.392 (T)	NCBI Database
India	*	0.270 (T)	G	0.070 (A)	NA	NA	[23]
Japan	T	0.450 (G)	G	0.147 (A)	NA	NA	[33]
Japan	T	0.497 (G)	G	0.137 (A)	C	0.102 (T)	[34]
Japan	T	0.460 (G)	G	0.143 (A)	NA	NA	[35]
Japan	T	NA	G	NA	NA	NA	[36]
Japan	T	0.493 (T)	G	0.123 (A)	NA	NA	[37]
Japan	T	0.450 (T)	G	0.194 (A)	C	0.124 (T)	[38]
Singapore (Chinese)	*	0.444 (G)	G	0.082 (A)	NA	NA	[39]
China (Beijing)	T	0.484 (G)	G	0.104 (A)	C	0.100 (T)	[25]
China (Xinjang Uygur)	G	0.188 (T)	G	0.047 (A)	T	0.244 (T)	present study
China							[40]
(Hong Kong)	NA	0.470 (G)	NA	0.124 (A)	NA	0.102 (T)	
(Beijing)	NA	0.497 (G)	NA	0.135 (A)	NA	0.084 (T)	
Asian (China)	NA	NA	NA	0.111 (T)	NA	0.067 (T)	
Asian (Japan)	NA	0.438 (G)	NA	0.125 (T)	NA	0.167 (T)	

NA indicates not available. The asterisk indicates that no association was found. MAF indicates minor allele frequency.

In the present study, alleles and genotypes were also significantly associated with XFS/XFG subjects between different gender and age groups. The alleles and genotypes in rs1048661 were not statistically significant between gender groups, as opposed to rs2165241 and rs3825942, which showed higher OR and 95%CI in the female group than in the male group. At rs1048661 the frequency of the G allele was higher in the patients than in the controls in the below 65-year-old group. At rs2165241 the frequency of the T allele was significantly higher in the patients than in the controls in both age groups. At rs3825942 the frequency of the G allele was significantly higher in the patients than in the controls in the over 65-year-old group. Therefore, gender and age might also play an important role in the pathogenesis of XFS/G in Uygur patients.

Although this is our first step in researching the polymorphisms of *LOXL1* in Uygur patients, the role of this SNP in pathogenesis of the disorder in Uygur patients needs to be studied further, and further investigations are also needed to determine additional genetic or environmental factors modifying the development of this disorder.
